# Preliminary molecular evidence associating a novel BRCA1 synonymous variant with hereditary ovarian cancer syndrome

**DOI:** 10.1038/s41439-018-0003-0

**Published:** 2018-04-20

**Authors:** Angelo Minucci, Paola Concolino, Maria De Bonis, Alessandra Costella, Ida Paris, Giovanni Scambia, Ettore Capoluongo

**Affiliations:** 1 Polo Scienze per Immagini, di Laboratorio e Infettivologiche, Teaching and Research Hospital “Agostino Gemelli” Foundation, Rome, Italy; 2 Department of Obstetrics and Gynecology, Division of Gynecologic Oncology, Teaching and Research Hospital “Agostino Gemelli” Foundation, Rome, Italy

## Abstract

Extensive molecular screening of the *BRCA1/2* (*BRCA)* genes by massively parallel sequencing (MPS) identified variants of uncertain (or unknown) significance (VUS) and novel variants. We performed a molecular characterization of a novel* BRCA1* synonymous variant discovered in a family with hereditary ovarian cancer (HOC) syndrome. We showed that the *BRCA1 c.5073* *A* > T variant might play a pathogenic role in HOC syndrome in this family.

Pathogenic variants (PVs) in the *BRCA1/2* (*BRCA*) genes predispose carriers to early onset hereditary breast and/or ovarian cancers (HBOCs).

A major limitation of *BRCA* testing, especially after adoption of the massively parallel sequencing (MPS)-based approach, is the increased number of uncertain (or unknown) significance(VUS) or novel variants. At the molecular level, a fraction of these variants occurs within the exon–intron boundary in highly conserved GT and AG dinucleotides at the 5’-intron or 3’-intron ends, respectively, resulting in either deletion of the exon or retention of the adjacent intron.

In this study, we report a 53-year-old Italian patient with a family history of high-grade serious ovarian cancer (onset age: 52-year-old) who was referred to our laboratory for *BRCA* testing (Fig. 1).

**Fig. 1:**
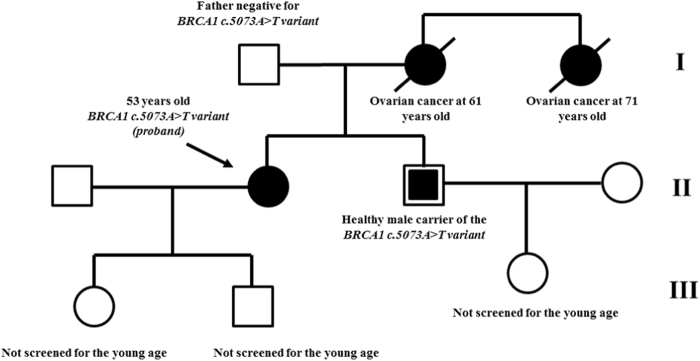
The pedigree of the proband’s family, cancer type, age onset, and MPS results are reported. We were unable to test other affected family members because these relatives had died. Moreover, we tested proband’s father to exclude the paternal origin of the variant and proband’s brother to enroll in a surveillance program for cancer prevention

The *BRCA* status obtained by MPS^1^ and MLPA (Multiplex Ligation-dependent Probe Amplification)^2,3^ did not reveal any known PVs. However, the patient carried a *BRCA1* variant in exon 17 (*c.5073* *A* > *T;* NCBI Reference Sequence: NM_007294.3; *GRCh37*; Fig. 2a). We considered the *c.5073* *A* > *T* variant to be novel because (a) we did not identify it among more than 4000 routinely analyzed alleles and (b) it was not found in the *Clin Var*. (https://www.ncbi.nlm.nih.gov/clinvar/), Ensemble (http://www.ensembl.org), Human Genome Mutation (www.hgmd.cf.ac.uk), ExAC (http://exac.broadinstitute.org), or 1000G (http://www.internationalgenome.org) databases.

**Fig. 2:**
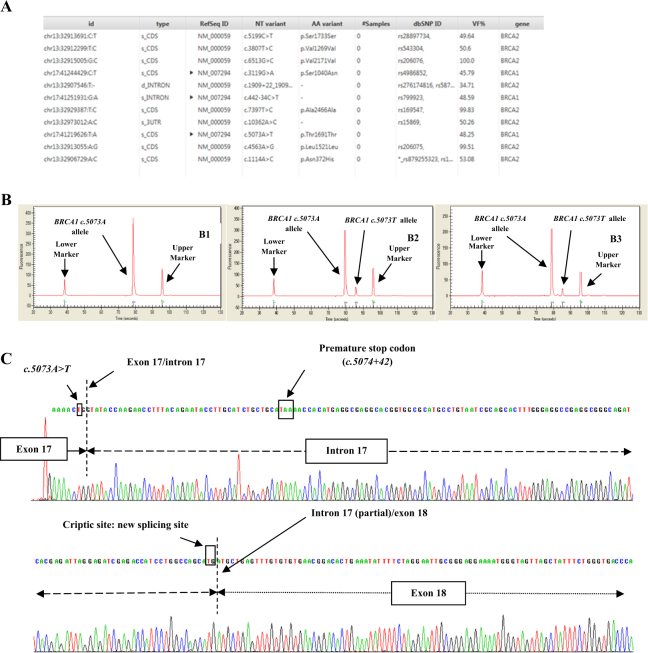
**a** Results of the proband’s genome obtained by MPS of the *BRCA* genes. Sequencing data, depth and variant calling were obtained by Amplicon Suite (Smart Seq, (Novara, Italy) software. **b** Capillary electrophoresis was used to check the amplification of the cDNAs of two women WT (**b**1) for the *BRCA1 c.5073A>T* variant, the proband (**b**2) and her brother (**b**3) carrier of the *c.5073A>T* variant. Arrows highlight the peaks corresponding to lower and upper markers and *c.5073A/T* alleles. In the control (**b**1) only the PCR product (499 bp) of the *c.5073A* allele is present. On the contrary, in the patient (**b**2) and her brother (**b**3), the mutated cDNAs gives two PCR products: the 499 bp band of the *BRCA1 c.5073A* allele and additional band of 652 bp due to intronic retention of 153 bp caused by the *BRCA1 c.5073T* allele. It is interesting to note that in both carriers, the proportion of the *c.5073T* allele is conserved (about 10% of the *c.5073A* allele). To verify the alternative splicing of the exon 17, Sanger sequencing was used **c**. The *c.5073T* allele causes the activation of a cryptic-splicing site resulting in a partial retention of 153 bp of intron 17. Moreover, we underline that the translation of the *c.5073T* allele stops after 42 bp (*BRCA1 c.5074+42*) due to the introduction of a stop codon (TAA codon) (as highlighted by the arrows)

In addition, family screening revealed the maternal origin of this variant and showed that the proband’s brother was a healthy carrier of the *c.5073T* allele (Fig. 1). All subjects gave their written informed consent for *BRCA* testing prior to blood sampling.

Several studies have shown that bioinformatics prediction tools can be used to prioritize variants for splicing assays^4^. The *c.5073* *A* > *T* variant involves the AG dinucleotides at the 3’ end of exon 17, and in silico analysis showed a high probability of pathogenicity due to the disruption of the WT splice donor, affecting the BRCT domain of BRCA1 (Supplementary Table 1).

For these reasons, to determine whether the *BRCA1 c.5073* *T* allele impaired the normal splicing process of exon 17, we analyzed the patient’s RNA. Total RNA was isolated from the patient’s leukocytes using the TRIzol® protocol (Thermo Fisher Scientific, Waltham, MA, USA) and retro-transcribed using the Transcriptor High Fidelity cDNA Synthesis Kit (Roche Diagnostics, Basel, Switzerland). cDNA was amplified using one primer pair that was specific to *BRCA1* exons 15–18 and another primer pair to demonstrate alternative splicing of exon 17. PCR was carried out using the Promega PCR Master Mix (Madison, WI, USA) with the following conditions: 95 °C for 2 min, 95 °C for 30 s, 60 °C (for the first pair) or 52 °C (for the second pair) for 30 s, and 75 °C for 1 min for 35 cycles with a final extension incubation at 72 °C for 10 min.

The amplified *BRCA1* WT and mutated alleles were distinguished by capillary electrophoresis on an Experion™ Automated Electrophoresis System (BioRad, Hercules, CA, USA) following the manufacturer’s instructions^5^.

All cDNA samples showed a fragment of 499 bp, corresponding to the expected amplification product of the *c.5073A* allele, confirming that they were successfully retro-transcribed. Moreover, an additional band longer than the WT allele appeared in the mutated cDNAs of two carriers (the proband and her brother) due to possible alternative splicing of exon 17 (Fig. 2b). To verify this hypothesis, a second amplification was performed. Analysis of the sequencing data showed that the *c.5073T* allele affected splicing reactions, resulting in the retention of the 153-bp intron 17 and allowing maintenance of the reading frame and possible translation of putative BRCA1 protein 51 amino acids longer than the WT protein. However, this splicing error introduces a stop codon (TAA) 42 bp after the *c.5073* *A* > *T* substitution (*c.5074* + *42*; Fig. 2c) terminating translation and results in a truncated protein.

Correct interpretation of the *BRCA* variants identified during MPS molecular testing is a critical step for definitive diagnosis, risk stratification of patients, and clinical decision-making. Integration of MPS with other molecular and cellular techniques to assess the significance of VUS or novel variants improves the sensitivity of *BRCA* testing overall, thereby leading to even more effective personalized medicine. This family of variants may also include synonymous variants in which a change occurs in the coding region of a gene but does not alter the amino acid sequence. In cancer, ~15% of synonymous variants have been estimated to cause human genetic diseases due to splicing defects^6^. Most frequently, the reported mechanisms are related to dysfunctional exonic splicing regulatory sites, such as enhancers and silencers. In this context, mRNA analysis, most preferably conducted on patients’ blood samples, can be used to verify the pathogenicity of these specific sequence variations.

In this study, we report the functional evaluation of a novel* BRCA1* splicing variant, *c.5073* *A* > *T* (p.Thr1691Thr). In silico analysis predicted that this variant exerted a pathogenic effect on normal splicing of *BRCA1* exon 17. Through analysis of total RNA, we demonstrated that the *c.5073* *T* allele caused an impairment of the normal splicing process of exon 17.

The results reported herein could explain the pathogenicity of this novel variant, particularly in view of the onset of ovarian cancer in this family.

In the Breast Cancer Information Core (BIC) database (https://research.nhgri.nih.gov/bic/) another variant (*c*.*5073* *A* > *G*, rs80356853, p.Thr1691Thr) has been reported and classified as VUS. Based only on the variant classification criteria by ENIGMA (Evidence-based Network for the Interpretation of Germline Mutant Alleles) rather than on functional studies, it has recently been re-classified as a “likely benign variant” (*Clin Var*. source). It is peculiar that the *c.5073* *A* > *G* (rs80356853) variant, for which bioinformatics analysis associates the same outcomes as the *c.5073* *A* > *T* variant, is a “likely benign variant”.

However, we underline that *BRCA1 c.5072* *C* > *T* results in aberrant splicing, whereas *c.5072* *C* > *A* has no effect on splicing^7^. We believe that further investigations are needed.

In conclusion, this case highlights the importance of studying the effects of novel* BRCA* variants at the mRNA level to verify their potential role in disease onset and to better define the HOC risk in the proband and family members.

## Limitations

It is generally accepted that variants resulting in a single major transcript lacking an open reading frame are deleterious^8^.

We emphasize that further evidence is needed to assign with certainty a pathogenic role to the *c.5073* *T* allele. In particular, it should be carefully evaluated whether the mutant allele causes both WT and aberrant transcripts or, alternatively, if the aberrant transcript is degraded by nonsense decay. In the same way, changes in the levels of low-abundance alternative splicing events could have an impact either directly or by altering the function or levels of endogenous transcripts, including full length mRNA. In addition, more evidence on the effects of the novel transcript on cellular function and the molecular interaction of WT BRCA1 with other associated genes is necessary. It will also be important to extend this investigation to ovarian tissue to gain a broader understanding of this splicing variant.

However, we believe that the use of cDNA studies is never conclusive. Multifactorial likelihood analysis methods that combine bioinformatics, pathologic, and clinical information are needed to definitively classify this type of  variants as pathogenic or benign.

 In conclusion, although this is preliminary data, we believe it is important to report these molecular evidence associated with the *BRCA1* *c.5073A>T* variant.

## Electronic supplementary material

Supplemental Material 1

## Data Availability

The relevant data from this Data Report are hosted at the Human Genome Variation Database at 10.6084/m9.figshare.hgv.1933.
