# Supplementary material for: Preliminary molecular evidence associating a novel BRCA1 synonymous variant with hereditary ovarian cancer syndrome
Source: Hum Genome Var. 2018 Apr 20;5:2. doi: 10.1038/s41439-018-0003-0 (PMC5938031; doi:10.1038/s41439-018-0003-0)
Supplement: Supplementary file 1 — Supplemental Material 1 [file 41439_2018_3_MOESM1_ESM.docx]

| *Exon* | *NT change*  *(HGVS)* | *Protein change* | *SpliceSite Finder*  *(0-100)*  *Th ≥ 70* | *MaxEntScan*  *(0-12)*  *Th ≥ 0* | *NNSplice*  *(0-1)*  *Th ≥ 0.4* | *Human splicing finder*  *(0-100)*  *Th ≥ 65* |
| --- | --- | --- | --- | --- | --- | --- |
| 17 | *c.5073A>T* | p.Thr1691Thr | SD: 71.90/NI  **(-100%)** | SD: 7.48/2.34  (**-68.6%)** | SD:0.92/NI  (**-100%)** | 76.86/72.11  (-6.2%) |

**Supplementary Table 1**. Splicing prediction analysis of the *c.5073T* allele

*NI*: not identified

**Supplementary Table 1**. Splice Site Finder (http://www.interactive-biosoftware. com), Splice Site Prediction by Neural Network (http://www.fruitfly.org/seq_tools/splice.html), MaxEntScan (http://genes.mit.edu/burgelab/maxent/Xmaxentscan_scoreseq.html), and Human Splicing Finder (http://www. umd.be/HSF/) were used to predict the effect of the *c.5073T* allele on the efficiency of splicing. Analysis was performed by the integrated software Alamut version 2.4 (http://www.interactive-biosoftware.com) using default settings in all predictions. A variation of more than 10 % in at least two algorithms was considered as having an effect on splicing.^14^The threshold (Th) represents the score above which a nucleotide position can be considered involving the splicing site. The scores indicate the value for splice donor (SD) of WT sequence (on the left) and mutated sequence (on the right). Significant scores (>10%) are reported in bold. In agreements with these criteria, the *c.5073A>T* variant was suggested to alter the donor splice site of the exon 17.
